# Bis(2-bromo­acetato-κ^2^ *O*,*O*′)(1,10-phenanthroline-κ^2^ *N*,*N*′)copper(II)

**DOI:** 10.1107/S1600536809009349

**Published:** 2009-03-19

**Authors:** Guofang He, Junshan Sun, Rengao Zhao, Jikun Li

**Affiliations:** a Department of Materials and Chemical Engineering, Taishan University, 271021 Taian, Shandong, People’s Republic of China

## Abstract

The two halves of the title compound, [Cu(C_2_H_2_BrO_2_)_2_(C_12_H_8_N_2_)], are related by twofold symmetry along the *b* axis through the central Cu^II^ ion. The Cu^II^ ion is coordinated by two symmetry-related N atoms from the 1,10-phenanthroline ligand and four O atoms from two 2-bromo­acetate ligands, showing a distorted octahedral geometry. Weak inter­molecular C—H⋯O inter­actions link neighbouring mol­ecules.

## Related literature

For a report on mononuclear, monomeric and polymeric metal complexes, see: Liu *et al.* (2006 ▶).
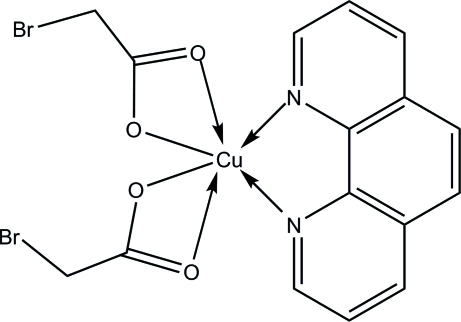


## Experimental

### 

#### Crystal data

[Cu(C_2_H_2_BrO_2_)_2_(C_12_H_8_N_2_)]
*M* *_r_* = 519.64
Monoclinic, 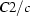

*a* = 10.3898 (16) Å
*b* = 17.974 (2) Å
*c* = 10.182 (3) Å
β = 116.142 (19)°
*V* = 1707.0 (7) Å^3^
*Z* = 4
Mo *K*α radiation
μ = 5.99 mm^−1^
*T* = 273 K
0.31 × 0.29 × 0.27 mm

#### Data collection

Bruker SMART APEX diffractometer
Absorption correction: multi-scan (*SADABS*; Sheldrick, 1996 ▶) *T*_min_ = 0.258, *T*_max_ = 0.295 (expected range = 0.174–0.199)
3953 measured reflections
1505 independent reflections
984 reflections with *I* > 2σ(*I*)
*R*_int_ = 0.066

#### Refinement

*R*[*F* ^2^ > 2σ(*F* ^2^)] = 0.057
*wR*(*F* ^2^) = 0.148
*S* = 1.00
1505 reflections
114 parameters
H-atom parameters constrained
Δρ_max_ = 1.11 e Å^−3^
Δρ_min_ = −0.78 e Å^−3^

### 

Data collection: *SMART* (Siemens, 1996 ▶); cell refinement: *SAINT* (Siemens, 1996 ▶); data reduction: *SAINT*; program(s) used to solve structure: *SHELXS97* (Sheldrick, 2008 ▶); program(s) used to refine structure: *SHELXL97* (Sheldrick, 2008 ▶); molecular graphics: *SHELXTL* (Sheldrick, 2008 ▶); software used to prepare material for publication: *SHELXTL*.

## Supplementary Material

Crystal structure: contains datablocks I, global. DOI: 10.1107/S1600536809009349/ez2160sup1.cif

Structure factors: contains datablocks I. DOI: 10.1107/S1600536809009349/ez2160Isup2.hkl

Additional supplementary materials:  crystallographic information; 3D view; checkCIF report

## Figures and Tables

**Table 1:** Selected bond lengths (Å)

Cu1—O2	1.941 (5)
Cu1—N1	2.016 (6)
Cu1—O1	2.725 (5)

**Fig. 1.**
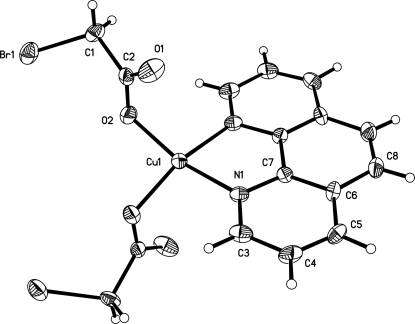
The molecular structure of title compound, with atom labels and 30% probability displacement ellipsoids for non-H atoms.

**Fig. 2.**
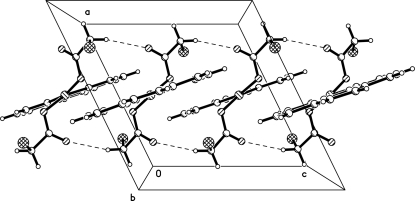
The stacking of the title compound into one-dimensional chains along the c axis. C—H···O interactions are indicated by dashed lines.

## supplementary crystallographic information

### Comment

Metal complexes with carboxylates are among the most investigated complexes in the field of coordination chemistry. Due to their versatile bonding modes with metal ions, they have been used in the synthesis of mononuclear, monomeric and polymeric complexes (Liu *et al.*, 2006). In order to develop new topological structures, we have studied the reaction of the copper(II) ion and 2-bromoacetic acid with the presence of 1,10-phenanthroline.

The molecular structure and a unit cell of the title complex are shown in Figs. 1 and 2. The Cu atom exhibits a six-coordinated distorted octahedral geometry. The two strongly bound carboxyl O atoms (Cu—O 1.941 (5) Å) and the two N atoms (Cu—N 2.016 (6) Å) occupy the equatorial positions. The two weakly bound O atoms (Cu1—O1 2.725 (5) Å) lie in the apical positions. Weak intermolecular C—H···O hydrogen bonds link the molecules into a one-dimensional chain structure along the c axis.

### Experimental

The reaction was carried out by the solvothermal method. 2-bromoacetic acid (0.104 g, 2 mmol) and cupric acetate (0.199 g, 1 mmol) and 1,10-phenanthroline (0.180 g, 1 mmol) were added to the airtight vessel with 20 ml water. The resulting green solution was filtered. The filtrate was placed for several days yielding blue block-shaped crystals. Yield: 78%. Elemental analysis: calc. for C_16_H_12_CuBr_2_N_2_O_4_: C 36.98, H 2.33, N 5.39; found: C 36.75, H 2.49, N 5.22. The elemental analyses were performed with PERKIN ELMER model 2400 series II.

### Refinement

All the H atoms were found in Fourier map, but placed in idealized positions (C—H 0.93–0.97 Å, O—H 0.85 Å), with the *U*_iso_(H) values set at 1.2*U*_eq_(C,O) of the parent atoms.

### Crystal data

**Table:**

[Cu(C_2_H_2_BrO_2_)_2_(C_12_H_8_N_2_)]	*F*(000) = 1012
*M**_r_* = 519.64	*D*_x_ = 2.022 Mg m^−^^3^
Monoclinic, *C*2/*c*	Mo *K*α radiation, λ = 0.71073 Å
*a* = 10.3898 (16) Å	Cell parameters from 1329 reflections
*b* = 17.974 (2) Å	θ = 2.5–25.5°
*c* = 10.182 (3) Å	µ = 5.99 mm^−^^1^
β = 116.142 (19)°	*T* = 273 K
*V* = 1707.0 (7) Å^3^	Block, blue
*Z* = 4	0.31 × 0.29 × 0.27 mm

### Data collection

**Table:**

Bruker SMART APEX diffractometer	1505 independent reflections
Radiation source: fine-focus sealed tube	984 reflections with *I* > 2σ(*I*)
graphite	*R*_int_ = 0.066
φ and ω scans	θ_max_ = 25.0°, θ_min_ = 2.5°
Absorption correction: multi-scan (*SADABS*; Sheldrick, 1996)	*h* = −12→12
*T*_min_ = 0.258, *T*_max_ = 0.295	*k* = −14→21
3953 measured reflections	*l* = −11→12

### Refinement

**Table:**

Refinement on *F*^2^	Primary atom site location: structure-invariant direct methods
Least-squares matrix: full	Secondary atom site location: difference Fourier map
*R*[*F*^2^ > 2σ(*F*^2^)] = 0.057	Hydrogen site location: inferred from neighbouring sites
*wR*(*F*^2^) = 0.148	H-atom parameters constrained
*S* = 1.00	*w* = 1/[σ^2^(*F*_o_^2^) + (0.08*P*)^2^] where *P* = (*F*_o_^2^ + 2*F*_c_^2^)/3
1505 reflections	(Δ/σ)_max_ < 0.001
114 parameters	Δρ_max_ = 1.11 e Å^−^^3^
0 restraints	Δρ_min_ = −0.78 e Å^−^^3^

### Special details

**Table:**

Geometry. All e.s.d.'s (except the e.s.d. in the dihedral angle between two l.s. planes) are estimated using the full covariance matrix. The cell e.s.d.'s are taken into account individually in the estimation of e.s.d.'s in distances, angles and torsion angles; correlations between e.s.d.'s in cell parameters are only used when they are defined by crystal symmetry. An approximate (isotropic) treatment of cell e.s.d.'s is used for estimating e.s.d.'s involving l.s. planes.
Refinement. Refinement of *F*^2^ against ALL reflections. The weighted *R*-factor *wR* and goodness of fit *S* are based on *F*^2^, conventional *R*-factors *R* are based on *F*, with *F* set to zero for negative *F*^2^. The threshold expression of *F*^2^ > σ(*F*^2^) is used only for calculating *R*-factors(gt) *etc*. and is not relevant to the choice of reflections for refinement. *R*-factors based on *F*^2^ are statistically about twice as large as those based on *F*, and *R*- factors based on ALL data will be even larger.

### Fractional atomic coordinates and isotropic or equivalent isotropic displacement parameters (Å^2^)

**Table:**

	*x*	*y*	*z*	*U*_iso_*/*U*_eq_	
Cu1	1.0000	0.05952 (7)	0.2500	0.0345 (4)	
N1	1.0486 (6)	0.1441 (3)	0.3940 (6)	0.0344 (14)	
O1	0.7140 (6)	0.0296 (3)	0.1339 (6)	0.0598 (17)	
O2	0.8977 (6)	−0.0134 (3)	0.0992 (6)	0.0486 (14)	
Br1	0.72014 (9)	−0.15682 (5)	−0.05512 (9)	0.0529 (4)	
C1	0.6633 (8)	−0.0541 (4)	−0.0643 (8)	0.0418 (19)	
H1A	0.6604	−0.0315	−0.1522	0.050*	
H1B	0.5675	−0.0517	−0.0705	0.050*	
C2	0.7655 (8)	−0.0101 (4)	0.0685 (7)	0.0344 (17)	
C3	1.0941 (7)	0.1419 (5)	0.5403 (8)	0.042 (2)	
H3	1.1074	0.0960	0.5866	0.051*	
C4	1.1215 (8)	0.2065 (5)	0.6232 (8)	0.045 (2)	
H4	1.1530	0.2031	0.7238	0.054*	
C5	1.1031 (7)	0.2740 (5)	0.5597 (8)	0.0416 (19)	
H5	1.1242	0.3170	0.6162	0.050*	
C6	1.0505 (7)	0.2786 (4)	0.4043 (8)	0.0336 (17)	
C7	1.0261 (7)	0.2126 (4)	0.3290 (7)	0.0277 (15)	
C8	1.0229 (9)	0.3460 (4)	0.3253 (9)	0.045 (2)	
H8	1.0354	0.3910	0.3746	0.054*	

### Atomic displacement parameters (Å^2^)

**Table:**

	*U*^11^	*U*^22^	*U*^33^	*U*^12^	*U*^13^	*U*^23^
Cu1	0.0365 (7)	0.0306 (8)	0.0303 (7)	0.000	0.0092 (6)	0.000
N1	0.038 (3)	0.042 (4)	0.020 (3)	0.001 (3)	0.010 (3)	0.002 (3)
O1	0.065 (4)	0.081 (5)	0.042 (3)	−0.013 (3)	0.031 (3)	−0.026 (3)
O2	0.044 (3)	0.036 (3)	0.055 (4)	0.000 (2)	0.012 (3)	−0.012 (3)
Br1	0.0681 (7)	0.0460 (6)	0.0436 (6)	−0.0113 (4)	0.0236 (5)	−0.0127 (4)
C1	0.048 (4)	0.050 (5)	0.022 (4)	−0.013 (4)	0.010 (3)	−0.009 (4)
C2	0.049 (5)	0.028 (4)	0.023 (4)	0.000 (3)	0.012 (4)	0.004 (3)
C3	0.039 (4)	0.060 (6)	0.030 (4)	0.001 (4)	0.017 (4)	0.012 (4)
C4	0.048 (5)	0.063 (6)	0.021 (4)	0.000 (4)	0.012 (4)	−0.011 (4)
C5	0.049 (5)	0.044 (5)	0.032 (4)	−0.008 (4)	0.018 (4)	−0.017 (4)
C6	0.044 (4)	0.025 (4)	0.037 (4)	−0.006 (3)	0.022 (4)	−0.006 (3)
C7	0.036 (4)	0.031 (4)	0.019 (3)	0.003 (3)	0.015 (3)	0.000 (3)
C8	0.056 (5)	0.030 (5)	0.046 (5)	0.001 (4)	0.020 (4)	−0.007 (4)

### Geometric parameters (Å, °)

**Table:**

Cu1—O2	1.941 (5)	C1—H1B	0.9700
Cu1—O2^i^	1.941 (5)	C3—C4	1.389 (11)
Cu1—N1	2.016 (6)	C3—H3	0.9300
Cu1—N1^i^	2.016 (6)	C4—C5	1.348 (11)
Cu1—O1	2.725 (5)	C4—H4	0.9300
N1—C3	1.350 (9)	C5—C6	1.431 (10)
N1—C7	1.368 (8)	C5—H5	0.9300
O1—C2	1.246 (8)	C6—C7	1.374 (9)
O2—C2	1.268 (8)	C6—C8	1.412 (10)
Br1—C1	1.929 (7)	C7—C7^i^	1.455 (12)
C1—C2	1.521 (10)	C8—C8^i^	1.392 (16)
C1—H1A	0.9700	C8—H8	0.9300
			
O2—Cu1—O2^i^	95.1 (3)	O1—C2—O2	124.6 (7)
O2—Cu1—N1	163.5 (2)	O1—C2—C1	118.5 (7)
O2^i^—Cu1—N1	93.4 (2)	O2—C2—C1	116.8 (6)
O2—Cu1—N1^i^	93.4 (2)	N1—C3—C4	121.6 (7)
O2^i^—Cu1—N1^i^	163.5 (2)	N1—C3—H3	119.2
N1—Cu1—N1^i^	82.1 (3)	C4—C3—H3	119.2
O2—Cu1—O1	53.88 (19)	C5—C4—C3	120.9 (7)
O2^i^—Cu1—O1	108.8 (2)	C5—C4—H4	119.6
N1—Cu1—O1	109.9 (2)	C3—C4—H4	119.6
N1^i^—Cu1—O1	87.6 (2)	C4—C5—C6	119.1 (7)
C3—N1—C7	117.5 (6)	C4—C5—H5	120.5
C3—N1—Cu1	129.3 (5)	C6—C5—H5	120.5
C7—N1—Cu1	113.1 (4)	C7—C6—C8	118.8 (6)
C2—O1—Cu1	72.7 (4)	C7—C6—C5	117.0 (6)
C2—O2—Cu1	108.7 (5)	C8—C6—C5	124.2 (7)
C2—C1—Br1	112.0 (5)	N1—C7—C6	123.9 (5)
C2—C1—H1A	109.2	N1—C7—C7^i^	115.8 (3)
Br1—C1—H1A	109.2	C6—C7—C7^i^	120.3 (4)
C2—C1—H1B	109.2	C8^i^—C8—C6	120.8 (4)
Br1—C1—H1B	109.2	C8^i^—C8—H8	119.6
H1A—C1—H1B	107.9	C6—C8—H8	119.6

Symmetry codes: (i) −*x*+2, *y*, −*z*+1/2.
